# Preparedness of health facilities in managing hypertension & diabetes mellitus in Kilimanjaro, Tanzania: a cross sectional study

**DOI:** 10.1186/s12913-019-4316-6

**Published:** 2019-07-31

**Authors:** Juma Adinan, Rachel Manongi, Gloria August Temu, Ntuli Kapologwe, Annette Marandu, Bahati Wajanga, Haruna Dika, Sarah Maongezi, Sweetness Laizer, Ridhiwani Manyuti, Rehema Abdillahi Nassir, Jenny Renju, Jim Todd

**Affiliations:** 1 School of AMO KCMC, 3010 Moshi, Kilimanjaro Tanzania; 2 Kilimanjaro Christian Medical University College, 2240 Moshi, Kilimanjaro Tanzania; 3 Department of Population Health, LSHTM, London, UK; 4 Catholic University of Health and Allied Sciences, 1464 Mwanza, Tanzania; 5 President’s Office - Regional Administration and Local Government (PORALG), Dodoma, Tanzania; 6 Ministry of Health, Community Development, Gender, Elderly and Children, Dodoma, Tanzania; 7 Mawenzi Regional Referral Hospital, Moshi, Kilimanjaro Tanzania; 8 Jaffery Charitable Dispensaries, Moshi, Kilimanjaro Tanzania

**Keywords:** Hypertension, Diabetes mellitus, Health facilities preparedness, Health services, Health system

## Abstract

**Background:**

Hypertension and Diabetes mellitus are risk factors for cardiovascular diseases that cause 17 million deaths globally. Many of these deaths could have been prevented if hypertensive and diabetic patients had their blood pressure and glucose controlled. Less than 30% of hypertensive and diabetic patients on management have controlled their blood pressure and glucose respectively. This study aimed to determine the preparedness of health facilities in managing hypertensive and diabetic patients in terms of personnel; laboratory services provision, and local use of routinely collected data, and shows differences in preparedness between the levels of facilities.

**Methods:**

We conducted a cross-sectional study in Government, faith-based and private health facilities in two districts in Kilimanjaro region in Tanzania from March to July 2017. We collected data through interviews and observations on the preparedness of the facilities for managing hypertension and DM.

**Results:**

Forty-three (43) health facilities and 62 healthcare workers (HCW) participated in the survey. Services for hypertension and DM were available in 37 (86%) and 34 (79%) health facilities respectively. Eighteen (53%) and five (15%) facilities had HCW trained on hypertension and DM management respectively within two years preceding the survey. Regular adherence to treatment guideline was reported in 18 (53%) of the health facilities. More than third of health facilities were without basic equipment for managing hypertension and DM. All the recommended laboratory tests were only available in four (15%) hospitals and one health center. Valid first line medicines for both hypertension and DM were available in six (50%) health centers, four (24%) dispensaries and in four (80.0%) hospitals. Health data collection, analysis and local use for planning were reported in all hospitals, nine (75%) health centers and four (24%) dispensaries.

**Conclusions:**

Health facilities are not fully prepared to manage hypertension and DM. Health centers and dispensaries are mostly affected levels of health facilities. Government interventions to improve facility factors and collaborative approaches to build capacity to HCW are needed to enable health facilities be responsive to these diseases.

## Background

Hypertension and diabetes Mellitus (DM) are common chronic diseases that are rapidly increasing in prevalence [1, 2]. Both diseases share lifestyle risk factors, require lifelong management, and pose life-threatening complications when not controlled [3–6]. Complications from both diseases lead to social and economic burden [2, 7, 8]. Hypertension and DM are both risk factors for cardiovascular diseases (CVD), which are responsible for 17 million (31%) deaths globally. Seventy five percent of all CVD associated deaths occur in developing countries [9].

In Tanzania, among adults over the age of 40 years, the prevalence of hypertension was 22–70% and the prevalence of DM was 2–8% with up to 70% of DM patients having hypertension comorbidity [10–15]. However, less than 30% of hypertensive and DM patients attending health facilities attained BP and glycemic control [10, 14, 16]. The increase in prevalence of hypertension-DM comorbidity necessitates evaluation of facilities preparedness for managing both hypertension and DM diseases.

All health facilities in Tanzania are expected to provide management services for hypertension and DM. Lower level facilities (dispensaries and health centers) are expected to refer complicated cases, but all health facilities should be prepared to provide health promotion, diagnosis and management services [17]. In addition, health facilities are supposed to have outreach programs to reach community members who cannot access health facilities [18]. To improve quality of care within the facilities, attending healthcare workers (HCW) should know and adhere to the treatment guidelines when managing patients with hypertension and DM [17]. HCWs are expected to correctly make diagnosis, screen for complications of these diseases, and provide appropriate treatment and advices to hypertensive and DM patients. Conducting imaging and laboratory investigations to screen for complications of these diseases facilitate patient-centered and so improves treatment outcomes.

Functioning health information systems are important for objective monitoring of the effectiveness of the health services in managing diseases, resources management, and as a measure of patient satisfaction [19–21]. Routinely collected data can be used to positively impact the health system through informing both clinical and managerial decisions. For example**,** managers can decide on equitable allocation of resources after identifying the real burden of the diseases, and HCWs can decide to whether or not modify management after analyzing treatment response of their patients.

Studies conducted in Tanzania and Uganda have documented a low level of preparedness of health facilities for the management of hypertension and DM [22–25]. However, these studies did not report on the laboratory services, adherence to treatment guidelines by HCWs and the functioning of the health management information system. Under the Improving Hypertension Management (IHM) Project we conducted studies to holistically evaluate health system preparedness in managing hypertension. This paper reports the preparedness of health facilities in managing hypertensive and DM patients in terms of personnel, laboratory services provision, and local use of routinely collected data, and shows differences in preparedness between the levels of facilities.

## Methods

### Study design and area

We conducted a cross sectional study in health facilities in two districts of Kilimanjaro region, Moshi District Council (MDC) and Moshi Municipal Council (MMC). We collected data from March to July 2017. MDC is a rural district, with area 1,300 km2 with a population of 466,737. MMC is an urban district with an area of 63.4 km2 and a population of 184,292 [26]. There were 138 health facilities in the two districts with 47 in MMC and 91 in MDC.

### Study population, sample size and sampling

All health facilities of all levels and all managing authorities were qualified for the study, but health facilities providing specialized services were excluded. A sample size of 43 (31%) health facilities was obtained to show the preparedness of the health facilities in managing hypertension and DM. Assuming that 75% of the facilities were prepared for the management of hypertension and DM, the study would have a power of 80% to show a difference of 20% between levels of facilities, as significant at the 5% level.

We selected health facilities from complete, accurate and up to date sampling frames obtained from the districts’ authorities. The first stage was stratification of the districts by geographical location and health facilities by the levels. The second stage was simple random selection of the health facilities from strata. Names of the facilities were entered in the MS. Excel program. Random numbers were then generated between one and the maximum number of the facilities per the stratum. Facilities were then selected consecutively until the allocated sample size for the strata was reached. Sampling unit was the health facility. The zonal and regional referral hospitals were included purposefully.

In each facility we randomly selected 62 HCWs who were attending outpatients on the days of data collection.

### Data collection and approach

We adapted the WHO service availability and readiness assessment (SARA) questionnaire to use for collecting data on availability of hypertension and DM services, and on the preparedness of health facilities to offer these services [26]. Trained research assistants interviewed facilities in-charges at the dispensaries and health centers. At the hospitals, the research assistants interviewed the facility in-charges and the head of the outpatient departments or hypertension and DM clinics. In addition, research assistants verified the equipment, medicine, medical supplies and health management information collecting tools that were reported to be available in each of the health facilities. Research assistants used Kobotoolbox**®** on tablets to collect the data. Kobotoolbox**®** is a free and open source online data collection software used by people working in humanitarian crises, as well as aid professionals and researchers working in lower and middle income countries [27].

We collected data on HCWs’ experience and confidence in managing hypertensive and DM patients by using a self-administered questionnaire. In addition, we collected data on HCWs’ practices towards advising patients on proper self-hypertension and DM management as well as identification of complications of these diseases.

### Study outcomes

Service provision was measured in two ways; hypertension and DM service provided at the health facilities and to the community during the outreach programs. We used six domains to evaluate preparedness: Trained staff and adherence to treatment guidelines by HCWs, availability of functioning basic equipment, medicines, laboratory services and health management information system and use (Table 1).

**Table 1:** Description of study variables. Description of main study variables and the methods used to collect them

Variable	Description
Service availability	
Service availability at the facility	Reported to provide diagnosis or treatment services for hypertensives and DM patients
Service provision to the surrounding catchment area	Percent of facilities reported visiting lower level health facilities or community within a catchment area of a respective health facility to provide DM and hypertension services
Trained personnel	Refresher training on managing hypertension and DM conducted within two years
Treatment guidelines	Reported adherence to treatment guidelines for management of hypertension and DM
Basic equipment	Observed functioning sphygmomanometer, stethoscope and Glucometer, weigh machine and height rod
Laboratory capacity	Availability of urine dipstick, creatinine, and cholesterol analysis
Medicine availability	Availability of at least one of the unexpired first line medicine for both hypertension and DM. For Hypertension: Bendroflumethiazide, Hydrochlorothiazide and spironolactone. For DM: Metformin & Glibenclamide
Health management information system०	Observed registers for routine data collection, reported analysis and local use of data for planning
Health Care workers (HCWs)	
Experience	HCWs attending ≥5 in three months was considered experienced
Confidence	Reported comfortable when managing hypertensive and DM patients
Practice on advice	HCWs’ reported to regularly advise hypertensives and DM patients on, dietary approaches to control these diseases, abandoning tobacco use, maintaining appropriate weight, exercising, and performing key investigations: performing tests to determine kidney function, cholesterol and chest x-ray or Echocardiogram

### Data management and analysis

Collected data were transferred to Stata V15 for data cleaning, processing and analysis. Furthermore, processing of graphs and tables was done using MS Excel 2015.

Variables were categorized to reflect Tanzania context. We merged all health facilities with “hospital status”, i.e. district hospitals, regional referral hospital and zonal referral hospital, into one hospital category, with health centers and dispensaries as separate categories. Furthermore, we merged ownership of the facility variable into two categories, public and private facilities from three categories public, faith-based and private.

Service availability for hypertension and DM was determined by dividing facilities reported to provide services for both hypertension and DM services by all 43 sampled facilities.

Preparedness was evaluated only in facilities that reported to provide both hypertension and DM services. A Facility was considered as prepared if reported to have all items/provide all services in the six domains we evaluated (Table 1).

We reported frequencies and percentages, and used the Chi square test to test the independence between categorical variables. A probability value (*P*-Value) of less than 5% was considered statistically significant. We presented study findings in tables and graphs.

## Results

### Description of the health facilities

The 43 health facilities included five hospitals (11.6%), 12(27.9%) health centers and twenty-six dispensaries (60.5%). Thirty-seven (86.0%) were public health facilities, and 26 (60.5%) were located in Moshi district council (Table 2).

**Table 2:** Facilities included in the study (*n* = 43). Percent distribution of Moshi Municipal and Moshi District Council health facilities by types, IHM project 2017

	Total (n = 43)	Hospitals (*n* = 5)	Health Centers (*n* = 12)	Dispensaries (*n* = 26)
Variables	n (%)	n (%)	n (%)	n (%)
Study area				
Moshi Municipal	17 [39.5]	3 [60.0]	5 [41.7]	9 [34.6]
Moshi District	26 [60.5]	2 [40.0]	7 [58.3]	17 [65.4]
Ownership				
Public	37 [86.0]	4 [80.0]	8 [66.7]	25 [96.2]
Private	6 [14.0]	1 [20.0]	4 [33.3]	1 [3.8]

Of the 62 HCW studied, 30 (48%) were clinical officers. Only at the dispensary level, nurses were attending hypertensive and diabetic patients (Table 3).

**Table 3:** Background characteristics of healthcare workers participated in the study (n = 62). Percent distribution of health care workers by types of facilities, IHM project 2017

Cadre	Total n (%)	Hospitals (*n* = 23) n (%)	Health Centers (*n* = 17) n (%)	Dispensaries (*n* = 22) n (%)
Clinical Officers (CO)	30 [48.4]	9 [39.1]	11 [64.7]	10 [45.5]
General physician	11 [17.7]	9 [39.1]	2 [11.8]	=0 [0]
Assistant Medical Officer (AMO)	8 [12.9]	5 [21.7]	4 [23.5]	0 [0]
Enrolled Nurse	4 [6.5]	0 [0]	0 [0]	4 [18.2]
Clinic Attendant	3 [4.8]	0 [0]	0 [0]	3 [13.6]
Registered Nurse	3 [4.8]	0 [0]	0 [0]	3 [13.6]
Nursing Assistant	3 [4.8]	0 [0]	0 [0]	2 [9.1]
Total	62 [100]	23 [100]	17 [100]	22 [100]

### Service availability

**Fig. 1:**
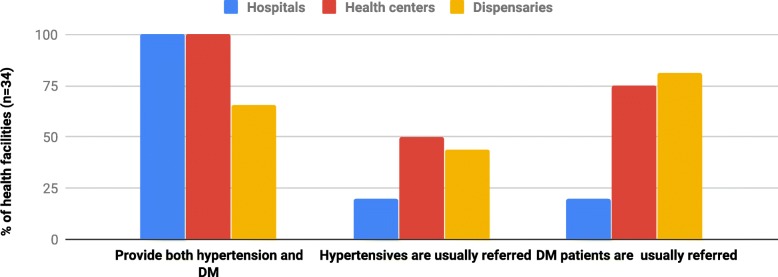
Percent of health facilities where services for hypertension and DM are available but regularly refer patients with these conditions (*n* = 43)

Hypertension and DM services were available in 37 (86.0%) and 34 (79.1%) facilities respectively. Facilities that were not providing HTN or DM services were all dispensaries. 27.0% (10/37) of the facilities that were providing hypertension services were regularly referring hypertensive patients while 41.2% (14/34) of the facilities that were providing DM services were regularly referring DM patients Fig. 1.

**Fig. 2:**
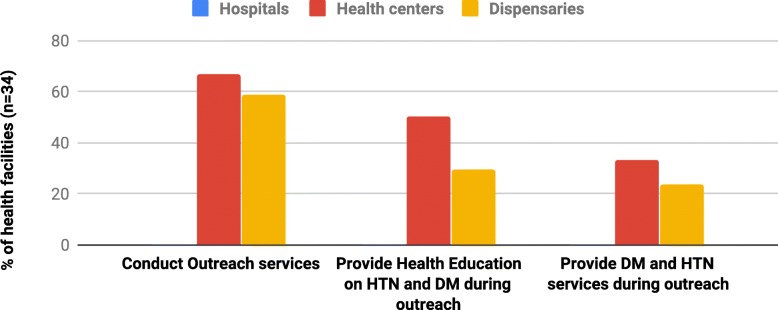
Percent distribution of health facilities that conduct outreach and provide DM and hypertension services during the outreach (*n* = 34)

Eighteen (41.9%) facilities, eight health centers and ten dispensaries, were conducting outreach services. Eleven (25.6%) of these facilities were providing health education on hypertension or DM. In addition, seven (16.3%) health facilities were providing hypertension and DM services during outreach Fig. 2.

### Preparedness of health facilities in providing management services for hypertension and DM

Eighteen (52.9%) facilities had HCWs trained on hypertension management. Six dispensaries and eleven health centers had HCWs trained on hypertension management. Five (14.7%) facilities had HCWs trained on DM management within two years preceding the survey: two were at the hospitals and the remaining three were at the dispensaries.

Adherence to treatment guideline by HCWs was reported in 18 (52.9%) health facilities. Regular adherence to guideline was reported in three (60.0%) of the hospitals, five (41.7%) of the health centers, and 10 (52.6%) of the dispensaries.

All basic equipment for managing hypertension and DM were available in 22 (64.7%) health facilities. All equipment was available in all hospitals, in eight (66.7%) health centers and in nine (52.9%) dispensaries.

All the recommended laboratory tests were available in four (14.7%) hospitals and one health center only. No dispensary had all the recommended laboratory tests available. The difference in the availability of the laboratory services between the levels of health facilities is statistically significant.

Valid first line medicines for both hypertension and DM were available in fourteen (41.2%) facilities. Six (50.0%) health centers and four (23.5%) dispensaries had all first line medicines compared to hospitals where four (80.0%) had all first-line medicines available (Table 4).

**Table 4:** Preparedness of health facilities in terms of Services provision for Diabetic and hypertensives by the types of health facilities (*n* = 43). Percent distribution of Moshi Municipal and Moshi District Council health facility preparedness in terms of availability of trained HCWs, treatment guideline, equipment, investigation, medicines and health management information system by types, IHM project 2017

	Total (=43)	Hospitals (*n* = 5)	Health Centre (n = 12)	Dispensaries (n = 17)	Chi^2^ *P*-Value
	n (%)	n (%)	n (%)	n (%)	
Personnel and guidelines					
HCWs trained in hypertension management	18 [52.9]	1 [20]	6 [50]	11 [64.7]	0.468
HCWs trained in DM management	5 [14.7]	2 [40]	0 [0]	3 [17.6]	0.306
Guideline for hypertension and DM observed	19 [76]	3 [60]	6 [66.7]	10 [76.9]	0.297
Adherence to guideline	18 [52.9]	3 [60]	5 [41.7]	10 [58.8]	0.622
Equipment					
Stethoscope	33 [97.1]	5 [100]	12 [100]	16 [94.1]	0.597
BP Machine	32 [94.1]	5 [100]	11 [91.7]	16 [94.1]	0.801
Glucometer	23 [67.6]	5 [100]	9 [75]	9 [52.9]	0.596
Weigh machine	32 [94.1]	4 [80.0]	11 [91.7]	17 [100]	0.090
Height rod	31 [91.2]	4 [80.0]	10 [83.3]	17 [100]	0.088
Facilities with all equipment	21 [61.8]	4 [80.0]	8 [66.7]	9 [52.9]	0.500
Laboratory tests					
Urine dipstick	30 [88.2]	5 [100]	11 [91.7]	14 [82.4]	0.000
Creatinine test	5 [14.7]	4 [80]	1 [8.3]	0 [0]	0.000
Cholesterol test	5 [14.7]	4 [80]	1 [8.3]	0 [0]	0.000
Facilities with all laboratory tests	5 [14.7]	4 [80]	1 [8.3]	0 [0]	0.000
Medicine availability					
Antihypertensive	18 [52.9]	4 [80]	6 [50.0]	8 [47.1]	0.417
DM medicines	23 [67.6]	5 [100]	10 [83.3]	8 [47.1]	0.030
Medicine	14 [41.2]	4 [80]	6 [50.0]	4 [23.5]	0.058
Health management information system					
Collection, analysis and local use of the data	18 [52.9]	5 [100]	9 [75]	4 [23.5]	0.002

Health data collection, analysis and local use for planning were reported in 18 (52.9%) facilities. All hospitals reported to use the collected data for planning compared to nine (75.0%) of the health centers and four (23.5%) of the dispensaries. The difference in the level of preparedness in terms of health data collection, analysis and use between the levels of health facilities is statistically significant (Table 4).

### Healthcare providers’ experience in, confidence about and reported practices of healthcare workers in managing hypertension and DM

Forty-three (69.4%) of HCWs were experienced in managing hypertensives and 14 (22.6%) were confident to manage hypertensive patients. Forty-seven (75.8%) HCWs wanted more training on hypertension management (Table 4). Twenty five (41.7%) of HCWs were experienced in managing DM patients and 13 (21%) were confident in managing DM. Forty eight (77.4%) HCW needed more training on managing DM.

HCWs were advising their patients to adhere to medications (93.3%), decrease salt intake (93.5%), not using tobacco and exercising (85.5%), regular check up of BP (83.9%) and blood glucose (83.6%) and attending appointments (87.1%). HCWs reported to advise their patients to do investigations for their kidneys (42.4%) and Chest X-Ray or ECG for their heart (37.3%). Statistically significant differences between the levels of health facilities on providing advise to patients are on reducing salt consumption, exercising, screening for DM and regular attendance to appointment (Table 5).

**Table 5:** Healthcare workers’ experience in, confidence about and reported practices in managing hypertension and DM patients (*n* = 62). Percent distribution of Moshi Municipal and Moshi District Councils’ HCPs knowledge, experience and comfortable in managing hypertension and DM patients by types of health facilities, IHM project 2017

	Total (*n* = 62)	Hospitals (*n* = 23)	Health centers (*n* = 17)	Dispensaries (*n* = 22)	Chi^2^ *P*-Value
Experience and knowledge of HCWs	n (%)	n (%)	n (%)	n (%)	n (%)
Experienced hypertension	43 [69.4]	23 [100]	10 [58.8]	10 [45.5]	0.000
Need more training for hypertension	47 [75.8]	16 [69.6]	12 [70.6]	19 [86.4]	0.000
Confident in caring for hypertensive	14 [22.6]	6 [26.1]	5 [29.4]	3 [13.6]	0.485
Experienced DM (*n* = 60)	25 [41.7]	20 [87.0]	4 [25.0]	1 [4.8]	0.000
Need more training for DM	48 [77.4]	16 [69.6]	11 [64.7]	21 [95.5]	0.000
Confident in caring for DM	13 [21.0]	6 [26.1]	6 [35.3]	1 [4.5]	0.094
Practices of HCWs					
Always advise patients on adherence (*n* = 60)	56 [93.3]	22 [95.7]	17 [100]	17 [85.0]	0.162
Always advise patients on lifestyle modifications (*n* = 60)	54 [87.1]	22 [95.7]	14 [82.4]	18 [81.8]	0.304
Always advise on not using tobacco	53 [85.5]	21 [91.3]	14 [82.4]	18 [81.8]	0.417
Always advise on physical activities/exercising	53 [85.5]	22 [95.7]	16 [94.1]	15 [68.2]	0.016
Always advise on reducing salt	58 [93.5]	23 [100]	17 [100]	18 [81.8]	0.021
Always advise to use plant-based cooking oil (*n* = 59)	43 [72.9]	17 [77.3]	12 [70.6]	14 [70.0]	0.237
Always advise on checking heart (*n* = 59)	22 [37.3]	10 [45.5]	5 [33.3]	7 [31.8]	0.778
Always advise on checking kidney (n = 59)	25 [42.4]	11 [47.8]	7 [50.0]	7 [31.8]	0.586
Checking for your blood glucose (*n* = 61)	51 [83.6]	22 [95.7]	15 [93.8]	14 [63.6]	0.007
Regular checking of blood pressure	52 [83.9]	21 [91.3]	15 [88.2]	16 [72.7]	0.314
Attending appointments	54 [87.1]	22 [95.7]	16 [94.1]	16 [72.7]	0.012

## Discussion

The study showed that hypertension and DM services are not available in 20% of facilities. Facilities that provide services have few trained HCWs, and the HCW have poor adherence to treatment guidelines. The facilities have incomplete sets of basic functioning equipment, lack laboratory tests for investigation services, experience first-line medicines stockout, and do not locally use routinely collected patients’ data for planning. In addition, HCWs are inexperienced and uncomfortable in managing diabetes and hypertensive patients, and a large proportion need more training.

Hypertension and DM services are unavailable in the majority of low-level health facilities. In addition, more than one third of the facilities that reported the provision of DM and hypertensive services, refer all these patients to other facilities for management of DM and hypertension (Fig. 1). Tanzanian health policies require all-levels of health facilities to provide basic management for hypertension and DM, and only refer when the conditions are complicated, so there is some way to go to achieve this requirement [18, 28]. Facilities providing hypertension services could reach the community around facilities not providing these services, however, Facilities providing hypertension and DM services during outreach programs are too few to address the existing and expected rise in burden of these diseases. Only nine of the thirty-four health facilities provide hypertension and DM services during outreach activities. Since majority of population seek initial medical services at the primary health facilities, service unavailability at the dispensaries and health centers may explain the low detection rate of and poor management outcome of hypertension and DM at the community [10, 14, 16]. In addition, these findings might explain poor health services utilization among community members as was reported elsewhere in Tanzania and other developing countries [24, 25].

Three-quarter of studied HCWs are inexperienced, not confident and needed training on management of hypertension and DM. The lack of confidence may explain routine referrals made by HCWs (Fig. 1). HCWs could use treatment guidelines to improve their confidence in managing hypertension and DM but treatment guidelines are either missing in majority of facilities or not followed where available (Table 4). Our findings are similar with those reported from similar health facility-based study conducted in Tanzania and Uganda [22, 24]. These findings call the responsible authorities and different health stakeholders to urgently identify training needs and train HCW as well as distribute treatment guidelines and advocate adherence to it.

Only five facilities are prepared to offer laboratory services. Hypertensive and DM patients need cholesterol, urine protein and creatinine [6, 17], to inform clinician’s decision on selecting effective management. The study showed that only five health facilities were prepared to offer these investigations, and four of these were hospitals. Referring patients for these investigations could allow proper evaluation for complications. However, less than 50% of healthcare workers routinely advice or refer patients to have these investigations (Table 5) when not available in their facilities. These findings imply that HCWs misses the opportunity of detecting hypertensive and DM complications at the early stages, and that we will continue to see patients presenting with complications while on management. Due to the advances in technologies, cheap rapid laboratory tests have been developed for glucose, cholesterol, creatinine and hemoglobin. The responsible authorities need to test the effectiveness of these devices in Tanzania and deploy those test that are effective. Deploying cheap rapid laboratory tests may address the unavailability of these key laboratory services.

Medicine stock out affects prescription of effective management [29] and health service utilization by patients [17, 23], and prohibits patient-centered management. In this study, metformin, which is recommended for obese diabetics is widely available, but glibenclamide, which is recommended for lean diabetics, is less widely available. Stock out of one type of medicine can be addressed by data-backed procurement. Medicine stock out has been reported to be a challenge in many developing countries including Tanzania [25, 29, 30].

Local use of routinely collected data is poor despite its usefulness in monitoring health facility resources and ensuring the effectiveness of the health system in managing and controlling diseases. Poor utilization of data leads to poor planning due to wrong estimation of the burden of diseases and missing feedback on the management outcome. All these impact negatively the effect of the facility to manage the diseases and wellbeing of patients. For example continuing prescription of ineffective management by HCWs after missing the fact that the regime does not work for their patients. None-use of the routinely collected data from hypertensive and DM patients may have both resulted from and contributed to low level of preparedness of health system in managing these diseases. This finding corroborates findings reported from similar studies elsewhere [31, 32], and calls for responsible authorities and other stakeholders actions to identify and address barriers to using routinely collected data.

### Strength and limitations

The strength of the study lies on the fact that the findings can be generalized to all types of facilities by ownership and levels. Involvement of all levels of health facilities with different ownership located in rural, semi-urban and urban localities allows broader understanding of the preparedness of the Tanzania health facilities in providing hypertension and DM services. Furthermore, assessment of HCWs practices, laboratory services provision and the local use of routinely collected data for planning add to the holistic evaluation of preparedness of the health facilities in managing DM and hypertension.

However, we studied HCWs who attend outpatients. At the hospitals settings these HCWs may not be representative of the HCWs attending inpatients. However, these results are still important as majority of hypertensive and DM patients are attended as outpatients. In addition, we determined the reported practices of HCWs who were managing hypertensive and DM patients. Self-reported practices of HCWs might have introduced information bias, however, we conducted exit interviews with patients on HCWs practices. Results on exit interviews will be reported in another publication.

The study findings are important as they support previously published findings and identify other critical challenges to improving hypertension and DM services. We evaluated essential components for the delivery of hypertension and DM services across a range of health facilities, which is needed if Tanzania is to provide the services to the whole population.

The study clearly shows where interventions are needed at the facility for outreach programs. It also identifies the training needs of HCWs in managing hypertension and DM and advocates for adherence to management guideline, in order to improve HCWs confidence in the delivery of hypertension and DM services. Deploying cheap and simple-to-use rapid tests will improve laboratory services and advocating for local data use will improve management services at the facilities’ and national level.

## Conclusion

Health facilities are not fully prepared to manage hypertension and DM. Health centers and dispensaries are mostly affected levels of health facilities. Government interventions to improve facility factors and collaborative approaches to build capacity to healthcare workers are needed to have health facilities responsive to these two ever-increasing diseases.

## Abbreviations

CVD: Cardiovascular diseases
DM: Diabetes Mellitus
HCWs: Healthcare workers
IHM: Improving hypertension management
MDC: Moshi district council
MMC: Moshi municipal council
